# Free fatty acids, not triglycerides, are associated with non-alcoholic liver injury progression in high fat diet induced obese rats

**DOI:** 10.1186/s12944-016-0194-7

**Published:** 2016-02-11

**Authors:** Jiali Liu, Lina Han, Leilei Zhu, Yerong Yu

**Affiliations:** Department of Endocrinology and Metabolism, West China Hospital, Sichuan University, Guoxue lane 37, Chengdu, Sichuan 610041 China

**Keywords:** Non-alcoholic fatty liver disease, Free fatty acids, Triglycerides, Oxidative stress, Lipid metabolism

## Abstract

**Background:**

The incidence of non-alcoholic fatty liver disease (NAFLD), commonly associated with obesity and metabolic syndrome, is increasing worldwide. However, the specific mechanisms that mediate the progression from simple steatosis to non-alcoholic steatohepatitis remain largely unclear. This study aimed to investigate the timedependent changes of triglyceride (TG) and free fatty acid (FFA) levels in the blood and liver over 24 weeks in high-fat diet-induced obese rats with NAFLD and to clarify the role of high FFA levels in the progression of liver injury.

**Methods:**

Male Wistar rats were randomly divided into three groups (*n* = 30 per group): the Control group, fed standard chow; the High-fat diet (HFD) group, fed high-fat chow; and the Acipimox group, fed an HFD plus acipimox (100 mg/kg/d, ig) for 8, 16 and 24 weeks. After treatment, blood and liver samples were collected for biochemical analyses, western blotting analysis and a histopathological study.

**Results:**

The visceral fat/weight and liver/body weight ratios were higher in both the HFD and Acipimox groups than in the Control group. The TG and FFA concentrations in blood and liver were increased in the HFD group and associated with elevated serum alanine aminotransferase (ALT) and liver malondialdehyde (MDA) levels and macro/microvesicular steatosis on hepatic fragments. Although the TG levels in the liver were similar between the HFD and Acipimox groups (*p* > 0.05), the FFA concentrations in the blood and liver were much lower in the latter group (*p* < 0.05). The Acipimox group showed normal ALT and MDA levels as well as less severe hepatic histological changes than did the HFD group (NAFLD activity score: 2.14 ± 0.14, 2.43 ± 0.20 and 2.63 ± 0.26 at 8, 16 and 24 weeks, respectively; *p* < 0.05 versus the HFD group at 24 weeks). The diacylglycerol acyltransferase 2 (DGAT2) protein levels were similar between the HFD and Acipimox groups (*p* > 0.05), but the protein expression level of carnitine palmitoyltransferase 1a (CPT-1a) was higher in the Acipimox group.

**Conclusions:**

Liver TG accumulation does not cause cellular injury in the liver; rather, FFAs or their metabolites are responsible for liver injury via increased oxidative stress. It is suggested that the therapeutic efforts to prevent non-alcoholic liver injury progression should be focused on reducing the burden of fatty acids transported to the liver or those being synthesized in the liver.

## Background

Non-alcoholic fatty liver disease (NAFLD) has rapidly become the most common cause of chronic liver disease worldwide. Clinical epidemiological data indicate that the prevalence of NAFLD ranges from 20 % to 30 % in Western countries and is 15 % in Asian countries [1]. NAFLD encompasses a spectrum of histological findings ranging from steatosis to steatohepatitis, and steatohepatitis can eventually progress to cirrhosis and even hepatocellular carcinoma [2, 3]. By some estimates, 10 ~ 20 % of patients with simple steatosis will convert into non-alcoholic steatohepatitis (NASH) [4] and 1.5 % to 3 % will develop advanced liver fibrosis [5], which seriously threatens the health of patients. Thus, to reduce the development of NASH and liver fibrosis/ cirrhosis, it is necessary to explore the pathophysiology and risk factors that influence the progression from simple fatty liver to NASH and to formulate early intervention strategies.

Although the model of the “two-hit theory” is widely accepted to explain the pathogenesis of NAFLD, the specific mechanisms that mediate the progression from simple steatosis to non-alcoholic steatohepatitis and those that lead the maintenance of simple steatosis in some patients are poorly understood. The presence of steatosis is a prerequisite for NASH, but it is not causal for the development of NAFLD. Some recent studies have found that lipid accumulation in the liver may be a protective mechanism for patients with obesity/insulin resistance/elevated free fatty acids (FFAs) [6, 7]. Hepatocytes, exposure to high levels of circulating FFAs, and stored FFAs in the form of triglycerides (TGs) may ease FFA-induced oxidative stress and chronic inflammation, thereby preventing further hepatocellular damage. However, whether elevated FFA levels in the bloodstream play a key role in the pathogenesis of NAFLD and whether lowering plasma and liver FFA levels can exert a protective effect on hepatocytes remains unclear.

Thus, to clarify the role of high FFA concentration in the progression of NAFLD, we induced NAFLD in rats using a high-fat diet and observed the time-dependent changes of TG, FFAs, liver enzymes, oxidative stress levels, and the protein expression levels related to TG synthesis and fatty acid oxidation. In addition, we utilized acipimox, a lipolysis inhibitor, to reduce blood FFA levels [8, 9] and determined whether reduced FFA levels had a protective effect on the liver.

## Methods

### Study animals and treatments

Eight-week-old male Wistar rats weighing 270 ± 10 g were obtained from the animal resources centre (Sichuan University, China). All of the protocols were approved by the Ethic Committee of West China Hospital, and the animals were handled according to the “Principles of Laboratory Animal Care.” The animals were maintained on a 12-h light/dark cycle with controlled room temperature (20 ± 2 °C) and had access to food and water ad libitum. After an adaptation period of 1 week, the rats were randomly divided into three groups: (1) the Control group (*n* = 30), fed standard rat chow; (2) the High-fat diet (HFD) group (*n* = 30), fed high-fat chow; and (3) the Acipimox group (*n* = 30), fed high-fat chow plus acipimox (100 mg/kg/day, ig). The percentages of grams and energy of the macronutrients in the diets are listed in Table 1. Body weight was measured weekly during the feeding period and compared with liver weight and visceral fat mass after the animals were sacrificed.

**Table 1:** Compositions of the chow and high-fat diets

	Chow diet		High-fat diet	
	g %	kcal %	g %	kcal %
Carbohydrate	60	67.4	21	15.8
Fat	4	10.1	39	66.1
Protein	20	22.5	24	18.1
Kcal/g	3.56		5.31	

At the end of 8, 16, and 24 weeks, 10 rats in each of the three groups were sacrificed under pentobarbital sodium anaesthesia. On the day prior to sacrifice, the rats were subjected to overnight fasting. Blood samples were obtained from the heart by cardiac puncture, and serum samples were preserved at −80 °C for biochemical analyses as described below. Liver samples were weighed and processed for biochemical and histopathological studies and western blotting analysis. Abdominal visceral fat (including perirenal, epididymal, and mesenteric fat) was collected and weighed.

### Blood biochemical analysis

The total cholesterol (TC) and TG levels were determined using enzymatic assays in commercial kits (Huili Biotechnologies Inc., Changchun, China). The FFA levels were assayed using the colorimetric method with a serum FFA kit (E1001, Applygen Technologies Inc., Beijing, China). The serum ALT activity, a biomarker for liver damage, was measured using an ELISA and a rat ALT kit.

### Assessments of hepatic TGs and FFAs

The liver TG (LTG) concentrations were quantified using a commercially available tissue TG kit (E1013, Applygen Technologies Inc., Beijing, China). Fatty acids were extracted from the liver using previously described methods [10, 11]. Briefly, 0.2 g of liver was weighed and placed into a manual glass homogenizer with lysis buffer (200 μL of 20 mM EDTA, 2 mM NaCl, and 50 mM sodium phosphate buffer, pH 7.4). Next, 10 μL of homogenate was mixed with 10 μL of tert-butyl alcohol and 5 μl of Triton X-100/methyl alcohol mixture (1:1 vol/vol) for lipid extraction [11]. The FFAs were measured using an enzymatic method. The protein concentrations in the liver extracts were determined using the BCA protein assay kit.

### Determination of malondialdehyde (MDA) and glutathione peroxidase (GSH-Px) in liver tissues

The levels of MDA in the liver tissue were determined using the thiobarbutaric acid (TBA) method with a commercially available kit (Jiancheng Bioengineering Institute, Nanjing, China), and the activity of GSH-Px was measured using the velocity method with a GSH-Px kit (Jiancheng Bioengineering Institute, Nanjing, China).

### Liver histopathology

Hepatic tissue was removed from each rat, and the same part of each liver was dissected, fixed in 10 % formalin, and then embedded in paraffin wax for staining with haematoxylin and eosin (H&E) and Masson’s trichrome. Coded sections were examined for histopathological changes by an experienced pathologist. Liver steatosis and NAFLD activity scores were semi-quantitatively evaluated as previously reported [12]. In brief, steatosis scores were defined as follows: score 0, presence of intrahepatic fat droplets in <5 % of hepatocytes; score 1, presence of intrahepatic fat droplets in 5–33 % of hepatocytes; score 2, presence of intrahepatic fat droplets in 33–66 % of hepatocytes; and score 3, presence of intrahepatic fat droplets in >67 % of hepatocytes. The NAFLD activity score is the sum of three equal weighted features: steatosis (0–3), lobular inflammation (0–3) and hepatocellular ballooning (0–2).

### Western blotting analysis

For the western blot analysis, 50 mg of hepatic tissue was homogenized on ice in radioimmunoprecipitation lysis buffer containing a complete protease inhibitor cocktail (Roche Diagnostics). The homogenates were centrifuged to collect the supernatants. The protein concentration was determined using a BCA protein assay kit (Thermo Scientific). Protein samples were subjected to SDS-PAGE and transferred onto polyvinylidene difluoride (PVDF) membranes. After being blocked with 5 % bovine serum albumin in PBST at room temperature for 2 h, the membranes were incubated with anti-DGAT2 antibodies (rabbit anti-DGAT2; 1:400; Santa Cruz Biotechnology) and anti-CPT-1a antibodies (rabbit anti-CPT-1a; 1:400; Santa Cruz Biotechnology) at 4 °C overnight. Subsequently, the membranes were washed three times with PBST and incubated with the relevant secondary HRP-conjugated antibodies (1:5000; ZSGB-BIO, Beijing, China) for 90 min. β-actin (mouse anti-β-actin; 1:500; ZSGB-BIO, Beijing, China) was used as the loading control. The intensity was measured using Quantity One densitometric software (Bio-Rad).

### Statistical analysis

All results are expressed as means ± standard error. Significant differences between the groups were determined using one-way analysis of variance (ANOVA) followed by Scheffe’s post hoc test using the SPSS 16.0 statistical package. A p-value <0.05 was considered statistically significant.

## Results

### Biometric parameters

As shown in Table 2, the body weight and visceral fat mass of the rats in each group increased with age. At 8 weeks, rats in both the HFD and Acipimox groups exhibited higher visceral fat mass and a higher visceral fat/weight ratio than did the age-matched control group, although no statistically significant differences were observed in body weight among the three groups. This phenomenon was more significant at the end of 16 and 24 weeks, when body weight, visceral fat mass, and the visceral fat/weight ratio in both the HFD and Acipimox groups were all markedly increased compared with the corresponding values in the Control group (*p* < 0.05). The liver/body weight ratio in the Control group did not change with time, whereas it was much higher in the HFD and Acipimox groups (*p* < 0.05). At 24 weeks, rats in the Acipimox group exhibited lower visceral fat mass and a lower visceral fat/weight ratio than did the rats in the age-matched HFD group, and no significant differences were observed between the HFD and Acipimox groups with respect to body weight or the liver/body weight ratio during the study period (*p* > 0.05).

**Table 2:** Weight, visceral fat, visceral fat/weight ratio, and liver/body weight ratio in the groups

	Control group			HFD group			Acipimox group		
Weeks	8 w	16 w	24 w	8 w	16 w	24 w	8 w	16 w	24 w
Weight (g)	419.7 ± 8.6	500.5 ± 9.8	582.6 ± 16.5	470.2 ± 17.8	646.2 ± 15.5^**^	690.5 ± 29.5^*^	456.0 ± 16.6	615.3 ± 22.2^**^	682.7 ± 20.2^*^
Visceral fat (g)	14.04 ± 0.79	17.43 ± 1.26	20.06 ± 1.78	24.71 ± 1.04^**^	52.97 ± 4.16^**^	73.44 ± 5.15^**#^	24.86 ± 0.67^**^	46.03 ± 4.22^**^	56.58 ± 3.39^**^
Visceral fat/weight (%)	3.5 ± 0.2	3.4 ± 0.3	3.6 ± 0.3	5.3 ± 0.3^**^	8.3 ± 0.7^**^	10.9 ± 0.5^**##^	5.5 ± 0.4^**^	7.5 ± 0.7^**^	8.7 ± 0.4^**^
Liver/body weight (%)	2.7 ± 0.1	2.5 ± 0.1	2.6 ± 0.1	3.2 ± 0.1^*^	3.3 ± 0.2^*^	3.1 ± 0.1^**^	3.1 ± 0.1^*^	3.1 ± 0.1^*^	3.0 ± 0.1^*^

Data are shown as means ± SEM. ^*^ *p* < 0.05 versus the corresponding Control group; ^**^ *p* < 0.01 versus the corresponding Control group; ^#^ *p* < 0.05 versus the corresponding Acipimox group; ^##^ *p* < 0.01 versus the corresponding Acipimox group; HFD: high-fat diet

### Serum biochemical analysis

As shown in Table 3, rats fed an HFD displayed higher circulating levels of TGs, TC, and FFAs than did the Control group rats. After 8 weeks, the serum TG levels in the HFD group increased approximately 3.4-fold compared to the Control group, whereas the FFA concentration only increased by 48 % in the HFD group; no significant difference was observed between the HFD and Control groups with respect to circulating TC levels. By 24 weeks, the HFD group showed a 2.2-fold increase in serum TG levels and a 56 % increase in serum TC levels, and the serum FFA concentration of the HFD group had increased to 2.7-fold that of the Control group. Acipimox treatment decreased the concentrations of TC, TG and FFAs compared to the HFD rats by 16 and 24 weeks (*p* < 0.05), and no significant differences were observed between the Acipimox and Control groups at any time point (*p* > 0.05) regarding the circulating lipid profile.

**Table 3:** Values of serum TC, TG, and FFAs in the studied groups

	Control group			HFD group			Acipimox group		
Weeks	8 w	16 w	24 w	8 w	16 w	24 w	8 w	16 w	24 w
TC (mmol/L)	1.05 ± 0.16	1.17 ± 0.15	1.24 ± 0.15	1.36 ± 0.08	1.81 ± 0.13^*#^	1.93 ± 0.17^*#^	1.18 ± 0.08	1.22 ± 0.11	1.21 ± 0.16
TG (mmol/L)	0.25 ± 0.04	0.30 ± 0.05	0.38 ± 0.05	0.84 ± 0.07^**##^	1.13 ± 0.14^**#^	0.84 ± 0.08^**#^	0.44 ± 0.06	0.65 ± 0.08	0.50 ± 0.07
FFAs (mmol/L)	0.29 ± 0.07	0.29 ± 0.07	0.34 ± 0.06	0.43 ± 0.07	0.90 ± 0.06^**##^	0.91 ± 0.10^**#^	0.34 ± 0.05	0.32 ± 0.07	0.56 ± 0.10

Data are shown as means ± SEM. ^*^ *p* < 0.05 versus the corresponding Control group; ^**^ *p* < 0.01 versus the corresponding Control group; ^#^ *p* < 0.05 versus the corresponding Acipimox group; ^##^ *p* < 0.01 versus the corresponding Acipimox group, *HFD*, high-fat diet, *TC*, Total cholesterol, *TG* triglycerides, *FFAs* free fatty acids

The serum ALT levels in the Control group remained stable during the study period (142 ± 2.3, 146 ± 4.4, and 144 ± 3.4 IU/L at 8, 16 and 24 weeks, respectively). The ALT level was 141 ± 3.7 IU/L in the HFD group at 8 weeks and increased progressively over time. By 16 and 24 weeks, the circulating ALT levels in the HFD group reached 155 ± 5 and 170 ± 4.0 IU/L, respectively. Compared to the Control group, HFD treatment caused an 18 % increase in the serum ALT level at the end of 24 weeks (*p* < 0.05). The serum ALT levels in the Acipimox group were 142 ± 2.6, 154 ± 4.4, and 150 ± 2.7 IU/L by 8, 16 and 24 weeks, respectively, and these findings were consistent with the levels observed in the Control group (*p* > 0.05).

### Hepatic TG and FFA contents

**Fig. 1:**
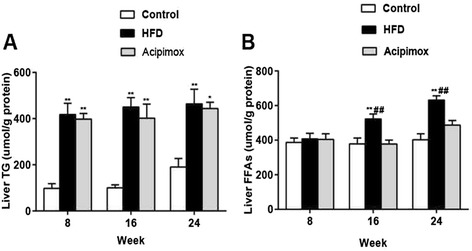
Time-dependent changes of liver TGs (**a**) and FFAs (**b**). Data are shown as means ± SEM; ^*^ *p* < 0.05 versus the corresponding Control group; ^**^ *p* < 0.01 versus the corresponding Control group; ^##^ *p* < 0.01 versus the corresponding Acipimox group; HFD: high-fat diet group; TGs: triglycerides; FFAs: free fatty acids

Both the HFD and Acipimox groups exhibited significantly higher liver TG levels than did the Control group. By 8, 16 and 24 weeks, the hepatic TG levels were 4.3-, 4.5- and 2.4-fold higher in the HFD group than in the Control group. No significant differences were observed in liver TG level between the Acipimox and HFD groups during the study period. The HFD treatment progressively increased the concentrations of hepatic FFAs over time. The liver FFA level in the HFD group increased by 38 % at 16 weeks and by 57 % at 24 weeks relative to the level in the Control group. Acipimox can effectively reduce the hepatic FFA content, and no difference in liver FFA level was detected between the Acipimox and Control groups at 8 and 16 weeks. By 24 weeks, the Acipimox group showed a slightly higher hepatic FFA content than did the Control group, but this difference did not reach statistical significance, and the hepatic FFA level in the Acipimox group was still significantly lower than that of the HFD group (Fig. 1).

### Changes in MDA and GSH-Px concentrations in the liver

As shown in Table 4, the concentration of hepatic MDA progressively increased in the HFD group over the 24 weeks. By 16 and 24 weeks, the liver MDA levels were 2.5- and 3-fold higher in the HFD group than in the Control group. Acipimox treatment markedly decreased the liver MDA levels compared with the HFD group (*p* < 0.05). After 16 weeks, the Acipimox group exhibited higher liver MDA levels than did the Control group, and no difference was observed between the Acipimox and Control groups regarding the hepatic MDA levels at 8 and 24 weeks.

**Table 4:** Values of hepatic MDA and GSH-Px in the studied groups

	Control group			HFD group			Acipimox group		
Weeks	8 w	16 w	24 w	8 w	16 w	24 w	8 w	16 w	24 w
hepatic MDA (nmol/mg)	1.59 ± 0.19	2.27 ± 0.30	2.41 ± 0.39	2.17 ± 0.21	5.65 ± 0.36^**#^	7.15 ± 0.51^**##^	2.01 ± 0.23	4.15 ± 0.28^**^	3.62 ± 0.52
hepatic GSH-Px (U/mg)	691.0 ± 31.2	674.1 ± 48.5	606.7 ± 36.2	471.5 ± 42.8^*^	463.0 ± 26.9^*^	504.4 ± 19.7	465.8 ± 56.4^*^	514.8 ± 45.4^*^	561.1 ± 54.6

Data are shown as means ± SEM. ^*^ *p* < 0.05 versus the corresponding control group; ^**^ *p* < 0.01 versus the corresponding Control group; ^#^ *p* < 0.05 versus the corresponding Acipimox group; ^##^ *p* < 0.01 versus the corresponding Acipimox group, *HFD* high-fat diet, *MDA* malondialdehyde, *GSH-Px* glutathione peroxidase

Rats in the HFD and Acipimox groups all displayed lower liver GSH-Px activity than did the Control group at 8 and 16 weeks (*p* < 0.05). After 24 weeks, no significant difference in hepatic GSH-Px activity was detected among the three groups.

### Changes in the TG synthesis and FFA oxidation rate-limiting enzymes in the liver

**Fig. 2:**
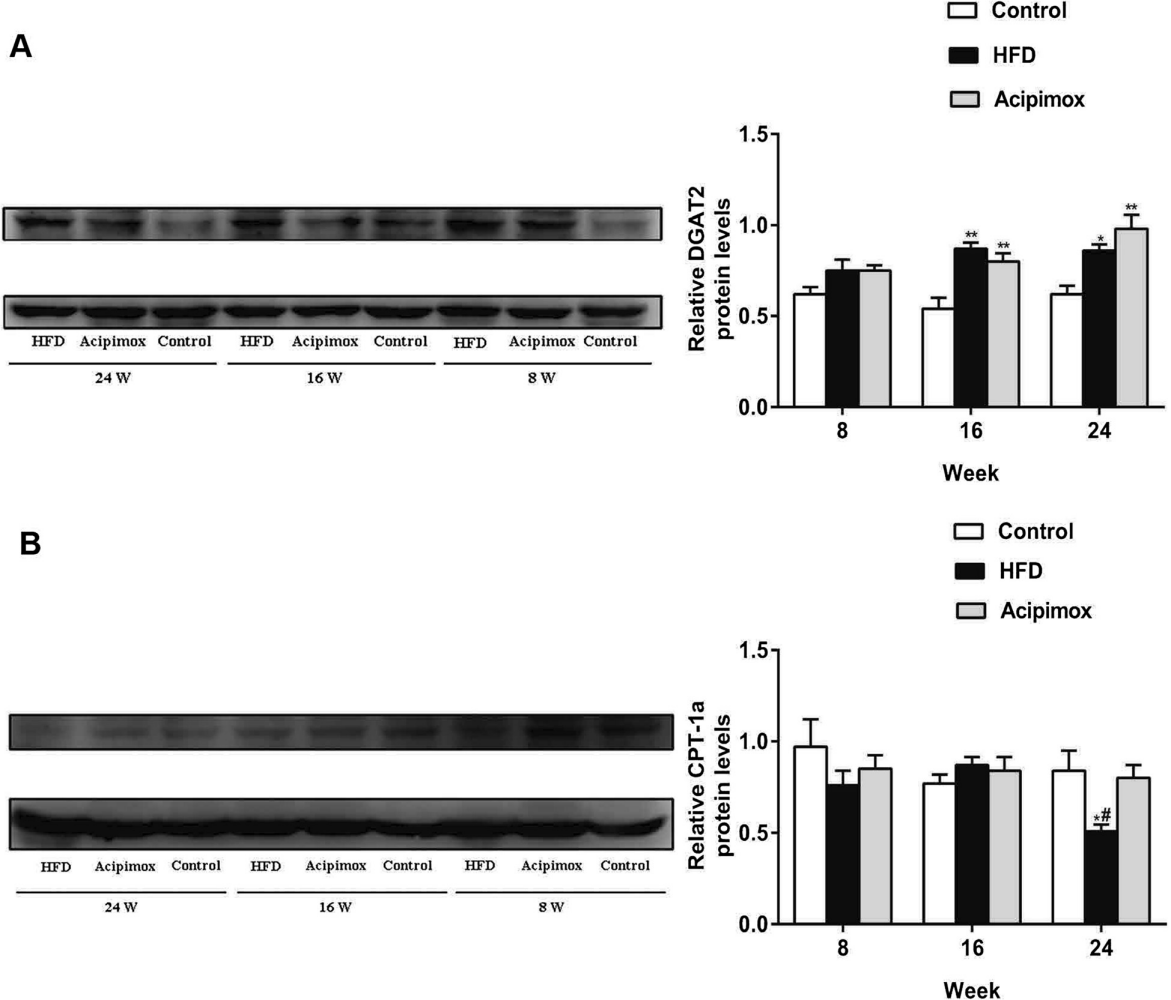
Hepatic protein expression levels of DGAT2 (**a**) and CPT-1a (**b**) in the studied groups. Left, protein level of DGAT2, CPT-1a and β-actin in rats liver treated as indicated. Right, densitometry quantification of DGAT2 and CPT-1a protein expression levels normalized to β-actin. Experiments were repeated three times. Data are shown as means ± SEM (*n* = 5-7). ^*^ *p* < 0.05 versus the corresponding Control group; ^**^ *p* < 0.01 versus the corresponding Control group; ^#^ *p* < 0.05 versus the corresponding Acipimox group; HFD: high-fat diet group; DGAT2: diacylglycerol acyltransferase 2; CPT-1a: carnitine palmitoyltransferase 1a

To understand the potential in vivo mechanisms that induced the changes in liver TG and FFA levels, we measured the hepatic protein expression levels of DGAT2, a TG synthesis rate-limiting enzyme, and the FFA oxidation-related enzyme CPT-1a. The HFD and Acipimox groups exhibited pronounced increases in DGAT2 protein level compared with the Control group. At the end of 16 and 24 weeks, the DGAT2 protein expression level had increased by 61 % and 39 % (HFD group) and by 48 % and 58 % (Acipimox group), respectively. No significant differences were observed in the DGAT2 protein level between the HFD and Acipimox groups at any time point. The protein expression level of CPT-1a was markedly reduced in the HFD group compared with the Control group at 24 weeks, although it did not differ significantly between the two groups at 8 and 16 weeks. The hepatic CPT-1a concentration can be effectively maintained by acipimox feeding and was similar between the Acipimox and Control groups (Fig. 2).

### Hepatic histological changes

**Fig. 3:**
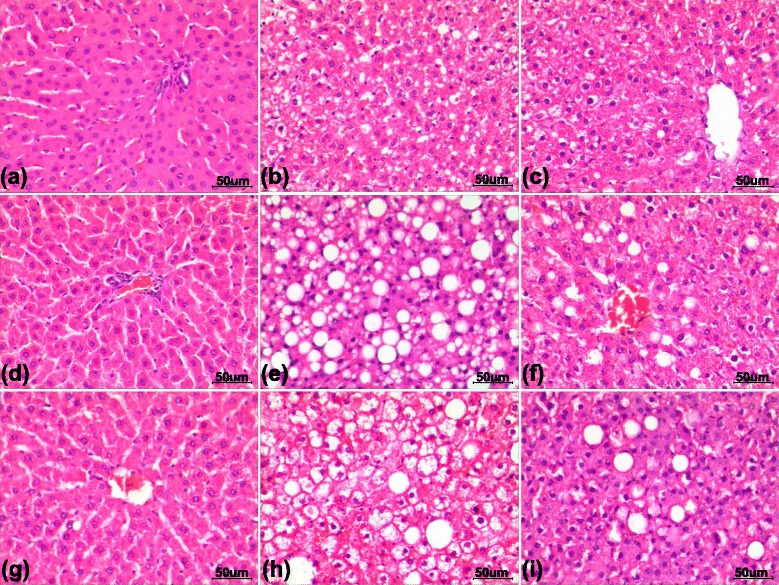
Liver sections stained with haematoxylin and eosin from rats fed with regular chow for 8 weeks (**a**), 16 weeks (**d**), or 24 weeks (**g**); from rats fed an HFD for 8 weeks (**b**), 16 weeks (**e**), or 24 weeks (**h**); or from rats fed an HFD + acipimox for 8 weeks (**c**), 16 weeks (**f**), or 24 weeks (**i**). Original magnification, 200×, and the length of the calibration bar is 50 um. HFD: high-fat diet

Representative histological images of H&E-stained liver sections are shown in Fig. 3. The HFD successfully induced hepatic steatosis, and HFD-fed rats displayed prominent hepatic steatosis (steatosis score: 2.25 ± 0.2) at the end of 8 weeks. At 24 weeks, the liver steatosis score was 2.6 ± 0.2 and was accompanied by ballooned hepatocytes and mild to moderate inflammatory cell infiltration. In the HFD group, the NAFLD activity score (NAS), a score that reflects the pathological severity of the liver, gradually increased from 2.3 ± 0.2 at 8 weeks to 3.5 ± 0.2 at 24 weeks (*p* < 0.05). The Acipimox-treated rats demonstrated HFD-induced hepatic steatosis (steatosis score: 2.1 ± 0.1 at 8 weeks and 2.4 ± 0.2 at 24 weeks), but ballooned hepatocytes were not observed in liver sections, and inflammation was less severe and less prevalent than that observed in the HFD group at 24 weeks (NAS: 2.6 ± 0.3; *p* < 0.05 versus HFD group). No rats in any treatment showed centrilobular or perisinusoidal fibrosis with Masson’s trichrome stain, and the liver sections of the Control group did not display any histological abnormalities.

## Discussion

At present, numerous dietary and/or genetic methods can be used to induce animal models, which can simulate the human condition of NAFLD in many aspects [13]. An HFD is one of these methods. In addition to hepatic steatosis, an HFD can often induce insulin resistance, lipid metabolism disorders, and other metabolic syndrome components with pathophysiology that is more similar to that observed in human NAFLD than that generated by other animal models of NAFLD [13, 14]. In this study, we fed eight-week-old male Wistar rats high-fat chow, and after 8 to 24 weeks of dietary treatment, the rats in the HFD group exhibited increased body weight, visceral fat mass, visceral fat/body weight ratio and liver/body weight ratio when compared with the levels in the age-matched control rats. HFD treatment also caused significant increments in the serum TC, TG level, elevated hepatic TG content and marked lipid droplet deposition in the liver sections, suggesting that the model of obesity accompanied by NAFLD was successfully induced in this study.

It is interesting to note that after 8 weeks of dietary treatment, the HFD group exhibited a 3.4-fold increase in serum TG level and a 4.3-fold increase in hepatic TG content compared with the levels in control rats. However, the increase in serum FFAs was only 48 %, and no significant change in hepatic FFA content was observed in HFD-fed rats compared with normal chow-fed rats. These findings suggest that for the first 8 weeks of the HFD, circulating FFAs were normally converted to TGs in the liver and were stored in adipose tissue, the liver, and other tissues, resulting in increases in body weight and fat mass. At this time, the liver enzyme levels were normal, although the liver/body weight ratio was increased and the liver tissue sections indicated microvesicular steatosis, which are consistent with signs of fatty liver.

As the HFD treatment progressed, this trend was reversed. By 24 weeks, the increase in serum TG level in the HFD group was only 2.2-fold that of normal rats, whereas the hepatic TG content was only increased 2.4-fold compared with normal rats. However, the serum FFA levels were 2.7 times higher and the liver FFA content increased by 57 % in HFD-fed rats compared with the Control group. We propose that the main mechanism for these changes at 24 weeks might be due to an impaired ability of the liver to synthesize TGs, resulting in significantly elevated serum and hepatic FFAs levels. In addition, at this time point, the serum ALT levels in the HFD group were elevated, and the NAS was 3.5 ± 0.2, which suggest the possibility of steatohepatitis. Several in vivo and in vitro studies have indicated that hepatic TG deposition may not be harmful; rather, it may represent a protective mechanism against FFA-induced lipotoxic liver injury by storing FFAs in the form of TGs [6, 7, 15]. To further study the role of an elevated serum FFA level on NASH, we treated the HFD-fed rats with acipimox to reduce plasma FFA levels by inhibiting lipolysis. These results showed that the serum FFA and TG concentrations in the Acipimox group were significantly lower than those in the HFD group. Although the Acipimox group did not significantly differ from the HFD group in liver TG content, the content of hepatic FFAs in the Acipimox group was consistent with that of the Control group. Despite the increase in the liver/body weight ratio, the Acipimox group did not show any obvious changes in serum ALT levels, and the NAS in the Acipimox group was also lower than that of the HFD group, indicating that acipimox treatment reduced liver damage in HFD-fed rats. These findings suggest that increased serum and liver FFAs concentrations rather than TGs may be a key link between obesity and the development of NAFLD.

An elevated circulating FFA level is one of the clinical characteristics of subjects with obesity/metabolic syndrome. Under conditions of insulin resistance, defects in the β-oxidation of FFAs are observed. Elevated FFA levels in the liver can cause hepatocyte injury via the formation of various toxic metabolites, such as ceramides, diacylglycerols, and lysophosphatidylcholine [16]. These metabolites can result in mitochondria dysfunction via multiple methods, such as inhibiting the mitochondrial oxidative respiratory chain [17], activating NADPH oxidase, or depolarising the mitochondrial membrane [18, 19], which can result in increased ROS production, thereby enhancing oxidative stress-induced liver injury. It has been shown that MDA, an index of oxidative stress, can damage cells and tissues, whereas GSH-Px, one of the body’s endogenous antioxidants, can protect hepatocytes against oxidative damage via chemical or enzymatic reactions. In our study, hepatic MDA levels were obviously elevated and progressively increased in the HFD group over 24 weeks compared with the Control group. Furthermore, the decrease in hepatic GSH-Px activity occurred at 8 weeks in the HFD group. The hepatic MDA level in the Acipimox group was similar to that of the control group and was markedly lower than that of the corresponding HFD group. Taken together, these findings suggest that a high serum FFA level may induce steatohepatitis by enhancing oxidative stress. When the level of FFAs is lowered with acipimox, hepatic oxidative stress levels decrease, thereby reducing liver damage and delaying the occurrence of NASH.

In the NAFLD state, the synthesis of hepatic TGs was increased. Although the classic “two hit” theory considers hepatic TG deposition to be a prerequisite for the development of NASH, emerging studies have suggested that TG accumulation may only be an “innocent bystander” or even a protective mechanism in the progression of NAFLD to NASH [7, 20]. In our study, the serum and liver FFA levels progressively increased in the HFD group over 24 weeks, but TG accumulation in the HFD group markedly decreased from 4.3-fold at 8 weeks to 2.4-fold at 24 weeks compared with the Control group, indicating that the ability of the liver to synthesize TGs is impaired with progression of the disease.

To further explore the potential mechanism that induces the change in liver TG content, we measured the hepatic protein expression levels of the TG synthesis-related enzyme DGAT2. DGAT2 is the rate-limiting enzyme in TG synthesis and plays an important role in the development of fatty liver diseases [21]. Previous studies have shown that the levels of DGAT2 mRNA/protein are elevated in the NAFLD state [22, 23]. The results of the present study are consistent with those of previous studies. Compared with the Control group, the HFD group exhibited a pronounced increase in DGAT2 protein level. Regarding the HFD group, no significant change in the expression of DGAT2 protein was observed during the course of the disease, and thus we speculate that an impairment of TG synthesis due to a decrease in DGAT2 protein expression was unlikely.

It is well known that CPT-1a is a gated enzyme that regulates mitochondrial β-oxidation in the liver. Previous studies have revealed conflicting results regarding the change in CPT-1a in NAFLD [24, 25]. The present study indicated that CPT-1a expression was similar between the HFD and Control groups at 8 weeks. By 24 weeks, CPT-1a expression was markedly decreased in HFD-fed rats. After treatment with acipimox, the level of CPT-1a protein expression was similar to that of the Control group, suggesting that during the progression of the disease, the oxidative capacity of mitochondrial fatty acids declines and that lowering hepatic FFAs levels may maintain the function of mitochondrial lipid oxidation, thus maintaining the balance of energy metabolism. We speculate that the decline in the oxidative capacity of mitochondrial lipids and oxidative stress-induced mitochondrial damage could jointly cause a decrease in ATP synthesis, resulting in an energy deficit in TG synthesis and subsequently impaired TG synthesis.

Finally, our study demonstrated that declining elevated serum FFAs levels were beneficial in preventing the conversion of simple steatosis to steatohepatitis. Nonpharmacologic interventions such as weight loss in obese patients, reduced carbohydrate intake, especially intake of high glycemic and high fructose foods, and aerobic exercise should be first-line choices. A number of agents, such as fibrates and nicotinic acid, can be used to reduce serum FFAs levels if nonpharmacologic interventions fail. In addition, nutraceuticals and functional food ingredients (such as fish oil and soyabean) that are beneficial to the lipid profile may reduce the risk of fatty liver by acting in parallel to pharmacologic therapy, as adjuvants in case of failure or in situations where pharmacologic therapy cannot be used [24, 26].

The main limitation of the current study is that we failed to induce NASH, mainly due to the limitations of the HFD-induced NAFLD model [13]. Although this drawback limits the extension of our results, we observed that the degree of liver damage was gradually increased as the HFD feeding period progressed via changes in NAS and serum ALT levels, and the hepatic FFA content and oxidative stress level showed pronounced changes. Therefore, we speculate that these changes should be more pronounced when the disease progresses to NASH.

## Conclusions

This study indicated that during the development of NAFLD, FFA levels increased significantly and were accompanied by an increase in oxidative stress. Then a reduction in FFA levels was observed, which can effectively reduce oxidative stress and liver injury. These findings suggest that although liver TG deposition is the primary characteristic of NAFLD, it may be an “innocent bystander” or a protective mechanism in the development of NAFLD. However, FFAs and their metabolites are likely, at least in part, the pivotal factor that promotes the development of NAFLD. Future studies are needed to obtain definitive results regarding this issue. If this is the case, then treatments should focus on how to effectively reduce the lipotoxicity of FFAs rather than inhibiting the deposition of hepatic TGs.

### Consent for publication

Not applicable.

### Open access

This article is distributed under the terms of the Creative Commons Attribution 4.0 International License (http://creativecommons.org/licenses/by/4.0/), which permits unrestricted use, distribution, and reproduction in any medium, provided you give appropriate credit to the original author(s) and the source, provide a link to the Creative Commons license, and indicate if changes were made. The Creative Commons Public Domain Dedication waiver (http://creativecommons.org/publicdomain/zero/1.0/) applies to the data made available in this article, unless otherwise stated.

## Abbreviations

NAFLD: Non-alcoholic fatty liver disease
NASH: Non-alcoholic steatohepatitis
HFD: High fat diet
TG: Triglycerides
FFAs: Free fatty acids
TC: Total cholesterol
MDA: Malondialdehyde
ALT: Alanine aminotransferase
GSH-Px: Glutathione peroxidase
DGAT2: Diacylglycerol acyltrasferase 2
CPT-1a: Carnitine palmitoyltransferase 1a
